# Your Grandchildren’s Pollen? Modeling the Future of Ragweed Sensitization in Europe

**DOI:** 10.1289/ehp.125-A60

**Published:** 2017-03-01

**Authors:** Charles W. Schmidt

**Affiliations:** Charles W. Schmidt, MS, an award-winning science writer from Portland, ME, writes for *Scientific American*, *Science*, various *Nature* publications, and many other magazines, research journals, and websites.

Many plants are growing faster, flowering earlier, and producing more pollen with rising atmospheric levels of carbon dioxide (CO_2_) and increasing surface temperatures.^1^ In this issue of *EHP*, scientists estimate how changing growth trends in ragweed may affect future pollen allergies in Europe, where the plant is a nonnative invasive weed. According to their modeled predictions, ragweed sensitization could more than double on the continent, from about 33 million people today to 77 million by the period 2041–2060.^2^

**Figure:**
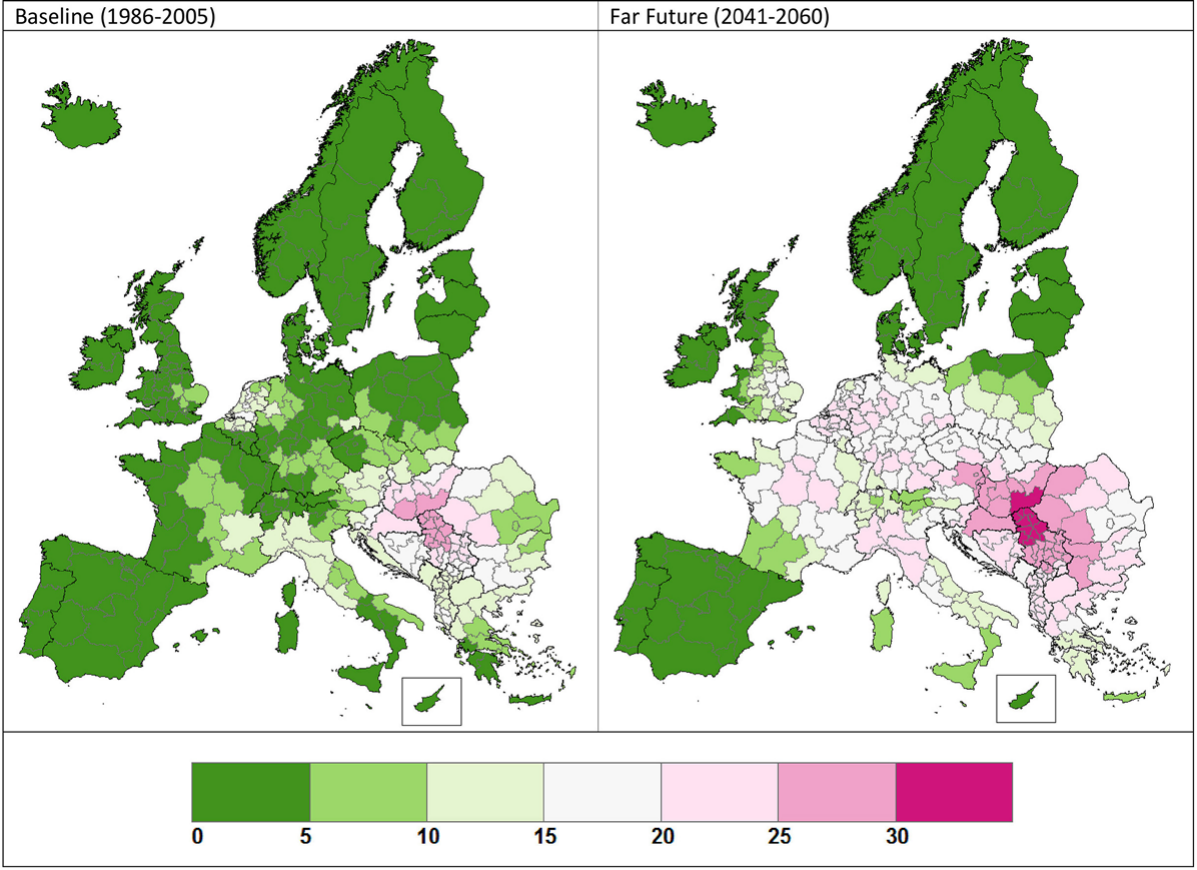
CO_2_ may be good for plant growth, but more growth also means more pollen. In a new study investigators estimated the percentage of the European population that will be susceptible to ragweed allergies in coming decades (the key in this figure indicates the estimated percentage of the population that is sensitized to ragweed pollen). Source: Lake et al. (2017)^2^

Sensitization means a person’s immune system produces antibodies against an allergen (in this case, pollen) and reacts to the allergen when re-exposed. Not all sensitized people develop allergy symptoms following re-exposure, but by one estimate, at least 60% of them do.^3^

CO_2_, a principal greenhouse gas, is the source of the carbon that plants need to make sugars during photosynthesis.^4^ ^,^ ^5^ Earlier research on ragweed plants showed that those raised in climate-controlled chambers grew larger and produced more pollen when CO_2_ levels were deliberately increased.^6^ Field evidence also indicates that warming temperatures and increased precipitation are lengthening the pollen seasons for allergenic plants in North America,^4^ ^,^ ^7^ even as ragweed’s range is expanding dramatically in Europe.^8^

The study was coordinated by the Atopica® project, which was a pan-European effort to explore the impacts of air pollution, land use, and climate change on pollen-induced allergic diseases. The project was launched in 2011 with funding from the European Commission and finished its activities in 2015.^9^

For the current analysis, the researchers relied on modeled projections of how ragweed’s range might expand in Europe under a variety of conditions.^10^ They used these projections to estimate future ragweed pollen levels for a 50 × 50-km grid across the continent under two greenhouse gas scenarios developed by the United Nations Intergovernmental Panel on Climate Change. These so-called Representative Concentration Pathway scenarios predict four possible atmospheric concentrations of greenhouse gases (expressed as CO_2_ equivalents) by 2100 based on emissions until then. The RCP4.5 scenario assumes that levels will reach 650 ppm by 2100 and stabilize thereafter, while the RCP8.5 scenario assumes that levels will reach 1,370 ppm by 2100 and continue to rise.^11^ The current atmospheric concentration of CO_2_ is just above 400 ppm.^12^

Finally, the authors linked projected pollen levels with information on ragweed sensitization and population density to estimate how many people would be sensitized to ragweed pollen in the future. Since estimates of future sensitization rates were similar under both scenarios until the middle of the twenty-first century (with rates diverging thereafter), the authors only reported data corresponding to RCP4.5.

Those results suggested that as ragweed plants produce more pollen, sensitization rates are likely to increase in areas where the plant is already endemic. But the biggest proportional increases will occur in places that currently do not have ragweed, says coauthor Michelle Epstein, an allergist in the Division of Immunology at the Medical University of Vienna: “Here we expect that climate change will allow ragweed to expand into new areas, such as Germany, Poland, and France.” She adds, “Our research suggests that ragweed pollen allergy will become a common health problem across Europe, expanding into areas where it is currently rare.”

Lewis Ziska, a research plant physiologist with the U.S. Department of Agriculture, describes the study as a “strong effort to link climate change, ragweed ecology, and health.” He adds, “As an invasive species, ragweed has no known enemies in Europe, so its ability to spread is limited mainly by climate.” Ziska was not involved in the study.

“The big message is that if we don’t do anything to mitigate climate change and its effects on ragweed growth, we’re going to have huge increases in allergy,” Epstein says. “In Europe, we expect it could reach epidemic proportions.”
